# Exploring RF Magnetron Sputtering Growth Composite Thin Film BiFeO_3_-Bi_2_Fe_4_O_9_ on C-Plane Al_2_O_3_ Substrate

**DOI:** 10.3390/ma16216987

**Published:** 2023-10-31

**Authors:** Suleiman Kallaev, Sadyk Sadykov, Anatoly Pavlenko, Mansur Ataev, Jiří Majzner, Farid Orudzhev, Kamal Giraev, Nariman Alikhanov

**Affiliations:** 1 Amirkhanov Institute of Physics, Dagestan Federal Research Center, Russian Academy of Sciences, St. M. Yaragskogo 94, 367003 Makhachkala, Russia; 2 Physical Department, Dagestan State University, St. M. Gadjieva 43-a, 367015 Makhachkala, Russia; 3 Federal Research Centre “The Southern Scientific Centre”, Russian Academy of Sciences, 344006 Rostov-on-Don, Russia; 4 Department of Physics, Faculty of Electrical Engineering and Communication, Brno University of Technology, Technická 2848/8, 61600 Brno, Czech Republic

**Keywords:** multiferroics, BiFeO_3_, Bi_2_Fe_4_O_9_, nanocomposite, film

## Abstract

Nanocomposite films of BiFeO_3_-Bi_2_Fe_4_O_9_ were fabricated on a sapphire substrate Al_2_O_3_ using the method of gas discharge high-frequency cathodic sputtering of a ceramic target with a stoichiometric composition in an oxygen atmosphere. The results of the film analysis using X-ray structural analysis, Raman scattering, XPS, and atomic force microscopy are presented. The lattice parameters, surface topography, chemical composition of the films, concentration, and average sizes of the crystallites for each phase were determined. It was shown that the ratio of the BiFeO_3_ to Bi_2_Fe_4_O_9_ phases in the obtained film is approximately 1:2. The sizes of the crystallites range from 15 to 17 nm. The optical and magnetic properties of the nanocomposite layers were studied, and the band gap width and magnetization hysteresis characteristic of ferromagnetic behavior were observed. The band gap width was found to be 1.9 eV for the indirect and 2.6 eV for the direct interband transitions. The magnetic properties are characterized by a hysteresis loop resembling a “wasp-waist” shape, indicating the presence of magnetic anisotropy.

## 1. Introduction

The increased interest of researchers in multiferroics, in which ferromagnetic and ferroelectric ordering coexist, is associated with the potential applications [1,2,3]. The connection between the ferroelectric and ferromagnetic or antiferromagnetic properties in magnetoelectric materials is widely used in multifunctional memory devices and spintronics [3,4]. One of the promising multiferroics is bismuth ferrite BiFeO_3_ (and its derivatives), which exhibits high temperatures of ferroelectric (T_C_ ≈ 1100 K) and magnetic ordering (T_N_ ≈ 645 K) [5]. At room temperature, the crystal structure has the *R*3*c* space group. The spontaneous polarization is oriented along the [111] direction of the pseudocubic perovskite unit cell, and the antiferromagnetic ordering of the G-type occurs such that the magnetic moments of the iron ions rotate in a spiral [101]. According to neutron diffraction data, the period of the cycloid is 62 nm [6]. Below the Neel temperature T_N_, bismuth ferrite has a complex, spatially modulated, magnetic structure of the cycloidal type, which does not allow for ferromagnetic properties. The destruction of its spatially modulated spin structure is a necessary condition for the occurrence of the magnetoelectric effect, which can be achieved by doping bismuth ferrite with rare-earth elements, creating nanostructured and thin film systems, or under the influence of high magnetic fields and pressures. Another way to suppress modulation is to create multiphase compounds. Recently, a new direction has emerged in condensed matter physics, studying multiphase systems consisting of compounds with different crystallographic structures and types of magnetic ordering, such as the composite BiFeO_3_-Bi_2_Fe_4_O_9_. The questions about the peculiarities of the formation of BiFeO_3_-Bi_2_Fe_4_O_9_ composites, the nature of the interaction, and the details of the mechanism linking their magnetic subsystems are currently widely discussed in the scientific literature. In [7,8,9], it is reported that nanocomposites based on the perovskite-like bismuth orthoferrite BiFeO_3_ and the mullite-like ferrite Bi_2_Fe_4_O_9_ demonstrate effects related to the exchange interaction at the interface of these phases.

Mullite Bi_2_Fe_4_O_9_, which is often observed as an impurity phase of BiFeO_3_ in the solid state, also belongs to the family of multiferroics [10,11]. Unlike BiFeO_3_, it has an orthorhombic crystal structure (space group *Pbam*) [12]. The magnetic properties of polycrystalline Bi_2_Fe_4_O_9_ depend on the synthesis method and the size of the crystallites. A sample with a crystallite size of 200–450 nm, synthesized using ethylenediaminetetraacetic acid [13], exhibits weak magnetization at room temperature. Polycrystalline Bi_2_Fe_4_O_9_ with a micron-sized grain, obtained by melting, undergoes an antiferromagnetic phase transition at 250–260 K [14,15]. Polycrystalline Bi_2_Fe_4_O_9_ ceramics with a grain size less than 200 nm are characterized by magnetic hysteresis at room temperature, which disappears upon heating [16].

In recent years, interest in bismuth ferrite has increased due to the discovery of a large magnetoelectric effect (ME) in BiFeO_3_-based films [17]. The observed ME is associated with the destruction of the spatially modulated structure in thin films, which is caused by high internal strain fields (due to the lattice mismatch between the film and substrate) [18]. Therefore, the development of technology for obtaining thin structures of multiferroic materials based on BiFeO_3_ on different substrates becomes a relevant direction.

It should also be noted that BiFeO_3_/Bi_2_Fe_4_O_9_ nanocomposites exhibit higher photocatalytic activity (compared to pure BiFeO_3_ and Bi_2_Fe_4_O_9_ individually) in the decomposition of rhodamine B and in the production of H_2_ from water under visible light. Such a heterostructure of BiFeO_3_/Bi_2_Fe_4_O_9_ and its synthesis strategy can provide new insights into the development of highly efficient photocatalysts [19].

This work presents the results of the synthesis and investigations of the structure, morphology, and optical and magnetic properties of a nanocomposite film BiFeO_3_-Bi_2_Fe_4_O_9_, synthesized on an Al_2_O_3_ sapphire substrate. To the best of our knowledge, there are few studies on thin films of Bi_2_Fe_4_O_9_ and BiFeO_3_-Bi_2_Fe_4_O_9_.

## 2. Materials and Methods

**Figure 1:**
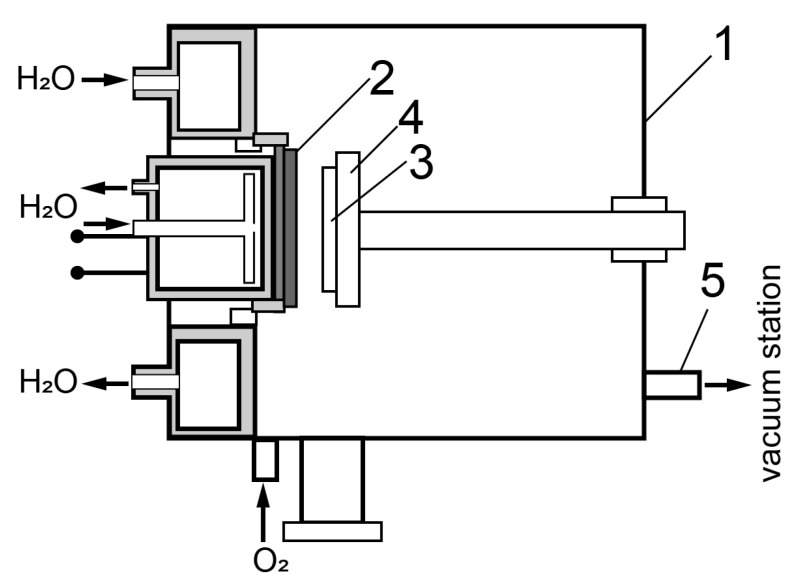
Scheme of the experimental setup for producing heteroepitaxial films. 1—Vacuum chamber, 2—ceramic target, 3—substrate, 4—ceramic heater, 5—output to vacuum station.

Gas discharge RF sputtering of BiFeO_3_ films was carried out on a “Plasma 50SE” installation (Manufacturer: ELITEH LLC, Moscow, Russia), designed for producing thin single-crystal films of complex oxides by reactive RF sputtering. The diagram of experimental setup for producing heteroepitaxial films is shown in Figure 1. Single-crystal Al_2_O_3_ (c-plane) 0.5 mm thick was used as a substrate. The initial temperature of the substrate is ~673 K, the oxygen pressure in the chamber is 80 Pa, the input RF power is 140 W. A ceramic disk of stoichiometric composition BiFeO_3_ with a diameter of 50 mm and a thickness of 3 mm was used as a sputtering target.

An X-ray diffraction pattern of a thin film sample on a substrate was taken on an XRD-7000 diffractometer from Shimadzu (Kyoto, Japan). The sample was placed on an attachment for studying thin films and shooting was carried out. The shooting parameters were as follows: tube voltage 30 kV; current 30 mA; X-ray wavelength λCuKα = 0.15406 nm; ray focusing scheme—grazing reflection geometry (grazing incidence X-Ray diffraction—GIXD); incident beam slit DS = 0.15°; graphite monochromator on a reflected beam; the angle of the incident beam Θ is fixed at value = 2°; scanning range −15–80°; scanning step −0.01°; scanning speed −0.2°/min; sample rotation speed −30 rpm. The measurements were carried out at room temperature T = 25 °C (298 K). The diffraction patterns were refined using Rietveld refinement via FULLPROF software (Version—July 2017).

Ntegra Spectra atomic force microscope was used to measure the surface topology of the as-grown samples and record Raman spectra (laser λ  =  532 nm). Magnetic measurements were carried out at room temperature using a vibrating sample magnetometer Lakeshore VSM 7404 (Lake Shore Cryotronics, Inc., Westerville, OH, USA). The chemical composition of the film was determined using energy dispersive X-ray spectroscopy (EDX-spectra, ASPEX Express, Delmont, PA, USA).

For measuring the optical properties were using an integrating sphere Avasphere-50 (Avantes, Apeldoorn, The Netherlands) in the wavelength range of λ~250–800 nm. The combined deuterium/halogen lamp AvaLight-DH-S-BAL (Avantes, Apeldoorn, The Netherlands) was used as the light source, and its emission was delivered to the samples via a 600 µm fiber optic light guide. Photonic signals were collected using a bifurcated 600 µm light guide and recorded with an MS3504i spectrometer (SOL-Instruments, Minsk, Belarus) combined with an HS-101(HR)-2048 × 122 CCD camera (Hamamatsu, Hamamatsu City, Japan) and a personal computer. The final data of the spectrophotometric coefficients *T_t_* and *R_d_* were defined as

(1) $$R_{d}^{exp}=\frac{R_{d}^{s}(\lambda )-R_{0}(\lambda )}{R_{gl}(\lambda )-R_{0}(\lambda )}\;\mathrm{and}\;T_{t}^{exp}=\frac{T_{t}^{s}(\lambda )-T_{0}(\lambda )}{T_{gl}\left(\lambda \right)-T_{0}\left(\lambda \right)},$$

where $T_{t}^{s}(\lambda )$ and $R_{d}^{s}(\lambda )$ are the transmission and reflection spectra of the samples; $T_{gl}\left(\lambda \right)$ and $R_{gl}(\lambda )$ are the reference signal spectra, measured with quartz plates; $T_{0}(\lambda )$ is the integrating sphere signal with covered input and open output ports; $R_{0}(\lambda )$ is the signal for a sphere with open optical ports. The calculation of the spectral dependence of the coefficients of optical absorption $\mu_{a}$ and light scattering $\mu_{s}^{\prime }$ was carried out using the inverse method of the Monte Carlo numerical simulation, developed and described in detail in the works [20,21,22].

Overview and region C1s and O1s X-ray photoelectron (XPS) spectra of the samples were collected on SPECS instrument (Specs GmbH, Berlin, Germany) using MgK-α excitation (Eex = 1254 eV). The recorded spectra were calibrated to pure graphite C1s energy (284.6 eV). XPS data were treated with CasaXPS software package (version 2.3.24, Casa Software Ltd., Teignmouth, UK). Shirley type background and mixed Gauss (70%)–Lorentz (30%) functions were used for the deconvolution of spectra under fixed FWHM for all spectral components.

## 3. Results

**Figure 2:**
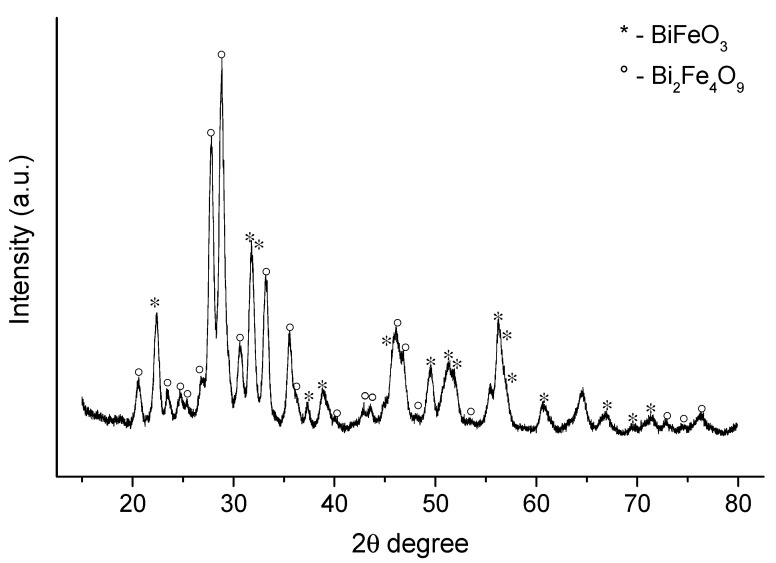
X-ray diffraction pattern of the BiFeO_3_-Bi_2_Fe_4_O_9_ film.

The results of the X-ray diffraction studies of a film on a leucosapphire Al_2_O_3_ (C-plane) substrate annealed at 550 °C for 2 h are presented in Figure 2. The analysis of the diffraction pattern showed the presence of peaks in good agreement with the standard declared values for BiFeO_3_ (ICSD # 98-015-7424, *R*3*c*), which can be attributed to rhombohedral planes with space group *R*3*c*. High intensity peaks related to Bi_2_Fe_4_O_9_ (ICSD #98-002-6808, *Pbam*) were also observed.

The volatile nature of bismuth may be responsible for the observed impurity phases. Previously, it was reported that during the synthesis process, the formation of stoichiometric compounds, such as Bi_2_Fe_4_O_9_ and Bi_25_FeO_40_, is inevitable [23,24,25]. According to previous research, this phenomenon is due to the product transformation during the crystal growth process [26].

The average crystallite size (*D*) was calculated using the Scherer formula:

(2) $$D=\frac{K\lambda }{\beta_{hkl}cos\theta }$$

where *K* is the shape factor (0.9), *λ* is the X-ray wavelength, *β_hkl_* is the full width at half maximum (FWHM) and *θ* is the Bragg angle. The calculation was carried out using the peak (012) (*θ* = 22.42°) for the BiFeO_3_ phase and (121) (*θ* = 27.793°) for the Bi_2_Fe_4_O_9_ phase, the average crystallite sizes were 14.9 nm and 17.3 nm, respectively.

**Figure 3:**
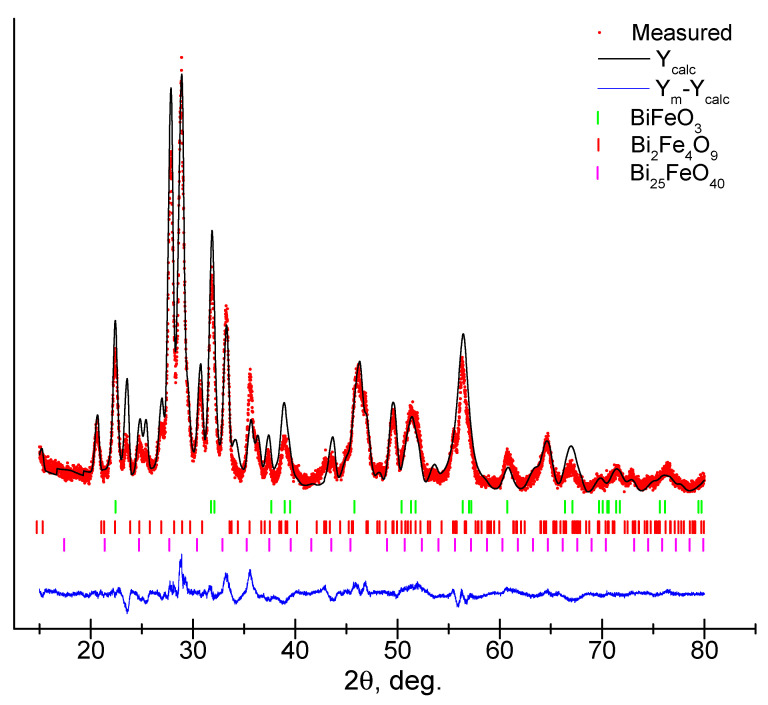
Refined X-ray diffraction pattern of a thin film using the Rietveld method.

The analysis of diffraction patterns using the Rietveld method shows that for the sample under study, the diffraction profile consists of the space group *R*3*c*, *Pbam*, and *I*23 with the contribution of BiFeO_3_ (33.4%), Bi_2_Fe_4_O_9_ (63.4%) and a small amount of Bi_25_FeO_40_ (3.2%) (Figure 3, Table 1). The content of Bi_25_FeO_40_ (ICSD #98-004-1937, *I*23) is quite small and within acceptable limits, and the contribution of its influence on the properties of the BiFeO_3_-Bi_2_Fe_4_O_9_ composite can be excluded. The refined lattice parameters and crystallite size are presented in Table 1.

**Figure 4:**
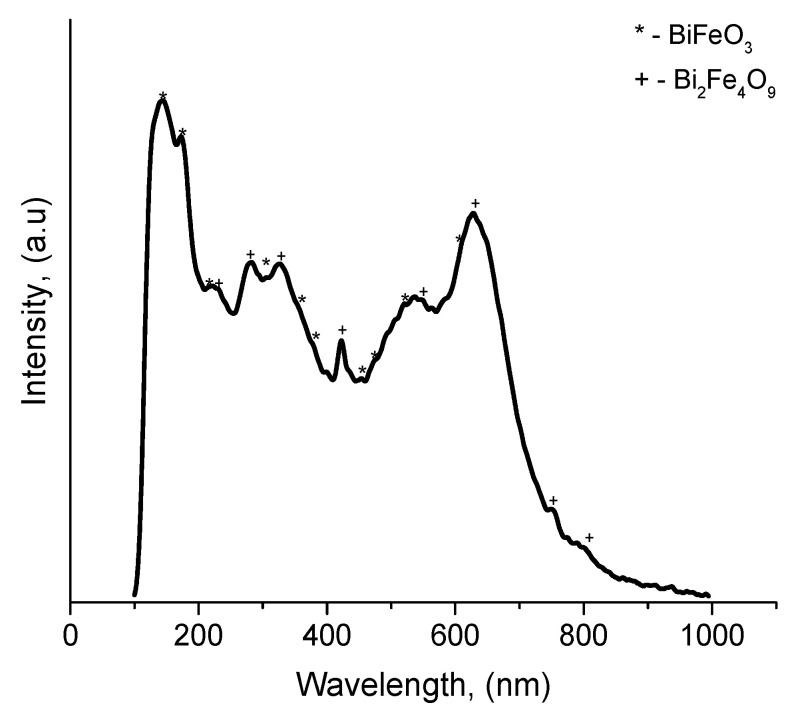
Raman spectra of BiFeO_3_-Bi_2_Fe_4_O_9_ film.

Raman scattering is one of the most powerful tools used to study structural features. Figure 4 shows the Raman spectrum in the range 100–1000 cm^−1^, showing the characteristic peaks expected for the BiFeO_3_ and Bi_2_Fe_4_O_9_ phases.

Note that for the compounds BiFeO_3_ and Bi_2_Fe_4_O_9_, despite being well-studied by Raman spectroscopy methods, there are discrepancies in the results in the literature. According to group theory [27,28], a rhombohedral BFO with space group *R*3*c* is characterized by thirteen (4A_1_ + 9E) active modes. Also, in [29], they showed that the mullite compound Bi_2_Fe_4_O_9_ is characterized by the presence of forty-two combination modes in the phonon spectrum out of a total number of ninety zone-center (Ӷ-point) modes in the Raman spectrum. However, as in the case of BiFeO_3_, for Bi_2_Fe_4_O_9_, in practice, a smaller number of phonon modes is observed compared to the above group-theoretic representation [28,30]. Table 2 shows the experimentally observed values of the Raman modes. The spectrum shows two distinct peaks at 143 and 175 cm^−1^ and eight weaker peaks in the range 218–609 cm^−1^. In the case of Bi_2_Fe_4_O_9_, clearly defined modes can be distinguished at 280, 328, and 423 cm^−1^. The observed Raman modes are in good agreement with the Raman spectra of BiFeO_3_ and Bi_2_Fe_4_O_9_ single crystals [29,31,32].

Thus, the results of the Raman analysis are in good agreement with the results of the X-ray diffraction analysis, confirming the coexistence of both rhombohedral BiFeO_3_ and orthorhombic Bi_2_Fe_4_O_9_.

**Figure 5:**
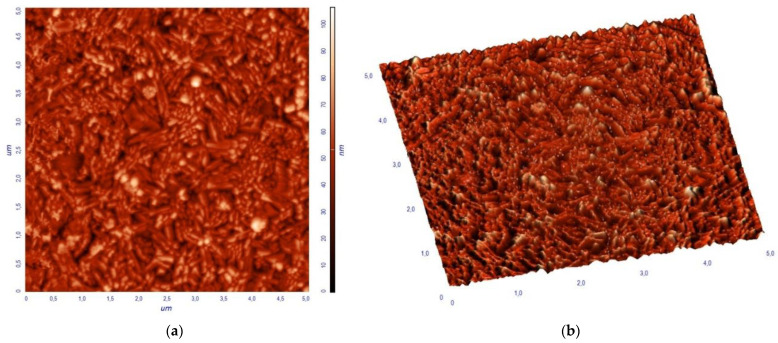
AFM images of the BiFeO_3_-Bi_2_Fe_4_O_9_ thin films: (**a**) 2D, (**b**) 3D.

The surface topography of a BiFeO_3_-Bi_2_Fe_4_O_9_ composite thin film with a scale area of 5 µm × 5 µm obtained by AFM is shown in Figure 5. The BiFeO_3_-Bi_2_Fe_4_O_9_ film has a dense surface without voids and cracks. The dense surface morphology and the absence of cracks and voids confirm the uniform deposition of thin films, suitable for the subsequent design of devices based on them. The analysis of the AFM image of the film shows that the grains on the surface are large compared to the size of their crystallites calculated using the Scherrer formula. In general, the grain size was in the range of 20–80 nm, and the average size was about 48 nm. The widely varying values of the crystallite size and grain size indicate that the latter are not single crystalline, but rather consist of many crystallites. The root mean square roughness (Rms) of the BiFeO_3_-Bi_2_Fe_4_O_9_ thin film was 11.14 nm.

**Figure 6:**
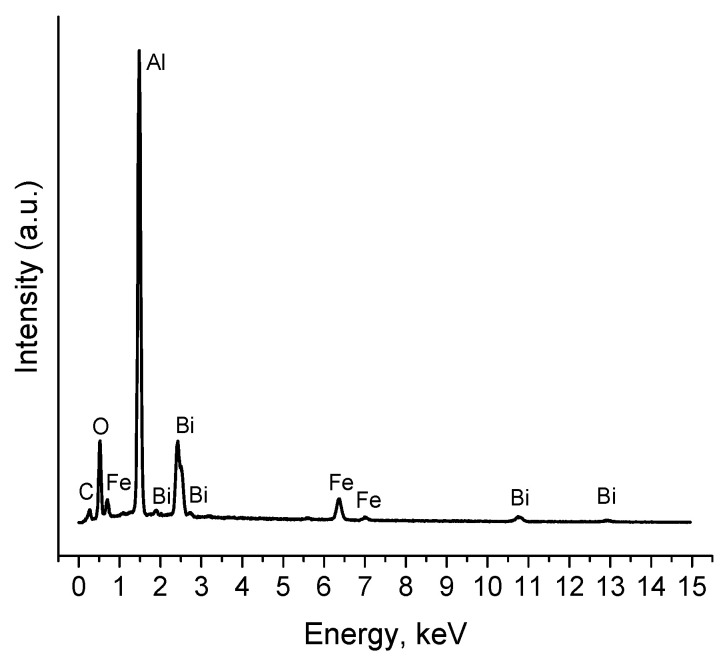
EDX spectrum of the BiFeO_3_-Bi_2_Fe_4_O_9_ film.

The results of studying the full energy spectrum of the BiFeO_3_-Bi_2_Fe_4_O_9_ composite film using energy dispersive X-ray spectroscopy (EDX) are presented in Figure 6. The elemental composition of the sample corresponds to the chemical formulas of the components and corresponds to the results of X-ray diffraction analysis. The EDX spectra were taken in several selected areas of the sample, and all showed the expected presence of Bi, Fe, and O. An Al peak associated with the Al_2_O_3_ substrate is also observed. The presence of a carbon peak is related to the research methodology.

**Figure 7:**
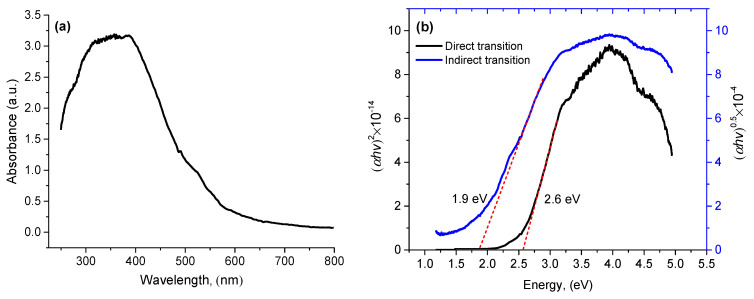
UV-visible spectra of a thin film (**a**) and Tauc plot of (*αhν*)^2^ and (*αhν*)^0.5^ versus *hν* for the optical band gap *E_g_* (**b**).

The optical properties of the BiFeO_3_-Bi_2_Fe_4_O_9_ nanocomposite were studied using UV-visible diffuse reflectance spectroscopy and converted into absorption spectra using the Monte Carlo method [20,22]. The absorption spectrum in the wavelength range 250–800 nm is shown in Figure 7.

As can be seen, the sample exhibits absorption in the ultraviolet and visible regions of light. The optical band gap (*E_g_*) can be estimated using the Tauc relation [33,34]:

(*αhν*) = K(*hν* − *E_g_*)^n^ (3)

where α is the absorption coefficient, *hν* is the photon energy, K is a constant, *n* is the coefficient characterizing the direct or indirect optical transition. The value of the band gap is determined by interpolating the linear part of the graph to the *X* axis. The linear regions on the Tauc plots (Figure 7b) indicate either an indirect optical band gap of 1.9 eV or a direct optical band gap of 2.6 eV. The existing literature indicates a wide band gap range (2.1–2.8 eV) for BiFeO_3_ and indicates the existence of both direct and indirect electron photoexcitation at room temperature in BiFeO_3_. However, the indirect charge transfer mechanism is thought to predominate at low room temperatures [35]. In the case of Bi_2_Fe_4_O_9_ films, the optical band gap is in the range of 2.05–2.2 eV [36,37,38]. From the theoretical studies of density functional theory [37], the band gap of Bi_2_Fe_4_O_9_ is considered to be indirect. For the Bi_2_Fe_4_O_9_-BiFeO_3_ sample, a band gap of 2.1 eV was observed [39]. Based on an analysis of the literature and the fact that the Bi_2_Fe_4_O_9_ phase predominates in our sample, we believe that in our case, an indirect optical transition occurs.

**Figure 8:**
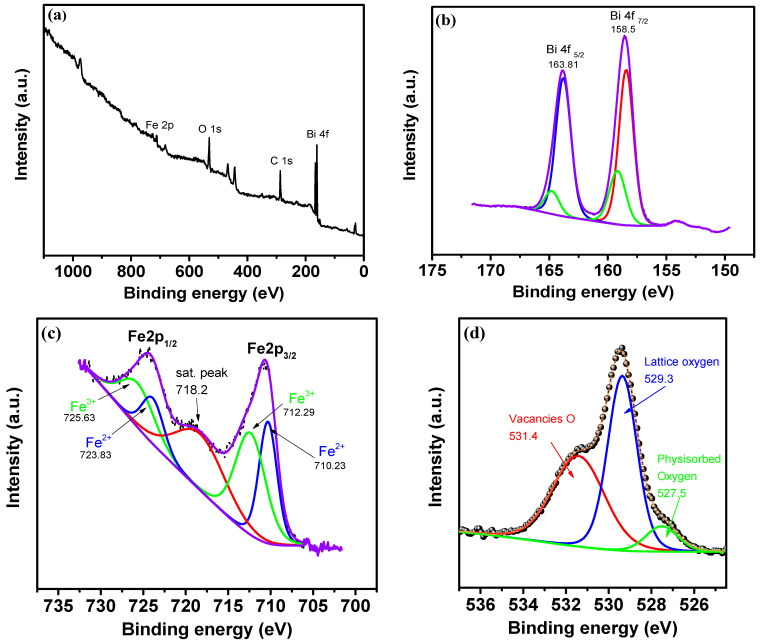
XPS spectra of BiFeO_3_-Bi_2_Fe_4_O_9_ composite film: panoramic spectra (**a**); Bi 4f (**b**); Fe 2p (**c**); O 1s (**d**).

Figure 8a shows the results of a panoramic scan of a thin film. Only C 1s, O 1s, Bi 4f and Fe 2p peaks were detected, indicating the absence of impurities, which is also confirmed by the EDX spectrum. The C 1s peak is due to carbon impurities [40,41]. Three regions associated with the Bi 4f, Fe 2p, and O 1s in the panoramic XPS spectrum of the sample were studied.

The high resolution Bi 4f spectra for the BiFeO_3_-Bi_2_Fe_4_O_9_ composite are shown in Figure 8b. The Bi 4f doublet consists of two peaks at 163.81 and 158.5 eV, which confirm the 3^+^ oxidation state of Bi, mainly representing the Bi-O bond. The spin-orbit splitting energy was Δ = 5.31 eV, which is comparable to the literature data [41,42,43] and is in good agreement with the theoretical value of 5.31 eV [44]. Two selected sub-peaks located at 163.74 and 158.31 eV are attributed to Bi (4f _5/2_)-O and Bi (4f _7/2_)-O bonds, while the other sub-peaks, located at 164.75 and 159, 11 eV, can be attributed to the Bi-O-Fe bond in the FeO_6_ octahedron, caused by oxygen vacancies and cation defects [45,46].

The spectra of the main level of Fe 2p are shown in Figure 8c. The asymmetric nature of the peaks and the presence of satellite peaks indicate the presence of iron in the oxidation states of Fe^2+^ and Fe^3+^. As shown in Figure 8c, the curve is divided into five components. The peaks of the Fe 2p doublets are concentrated at 724.31 eV (Fe ^3+^ 2p_1/2_) and 710.63 eV (Fe^3+^ 2p_3/2_), which represent Fe-O bonds [45]. The spin-orbit splitting energy of pure Fe 2p is 13.68 eV, which is close to the theoretical value (Δ Fe 2p) of 13.6 eV for Fe_2_O_3_ [45]. The peaks of the Fe 2p doublets located at 725.63 eV and 712.29 eV can be attributed to the (Fe 2p_1/2_)_2_–O_3_ and (Fe 2p_3/2_)–O_3_ bonds, and the subpeaks located at 723.83 and 710.23 to the bonds Fe-O, Fe-O-Bi bonds in the FeO_6_ octahedron, oxygen vacancies, and cation defects [47].

According to the ratio of the fitted peak areas for Fe^3+^ and Fe^2+^, the concentration ratio of Fe^3+^ and Fe^2+^ is approximately 1.43, indicating a relatively high Fe^2+^ content, which in turn may affect the magnetic properties of the composite.

To confirm the presence of oxygen vacancies, the spectrum of the O 1s level was studied. Figure 8d shows the O 1s spectra after deconvolution with the approximation of the fitted peaks. The spectrum is well-described by the superposition of three components centered at 527.5, 529.3, and 531.4 eV, respectively. A strong peak at 529.3 eV is assigned to the characteristic signal from lattice O. The shoulder at about 531.4 eV belongs to defective O components, such as oxygen vacancies, and the peak at 527.5 to physisorbed oxygen [42].

**Figure 9:**
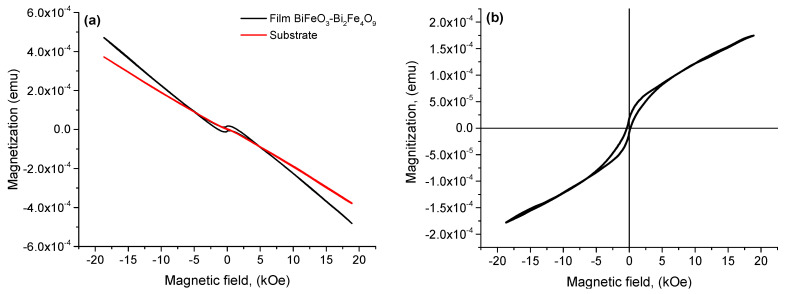
M−H magnetic hysteresis loops before (**a**) and after (**b**) subtraction of the diamagnetic contribution of the substrate.

Figure 9a shows the magnetic hysteresis loops M–H of the BiFeO_3_-Bi_2_Fe_4_O_9_ composite structure, measured at room temperature in magnetic fields up to 20 kOe. Figure 9b shows the M–H loop considering the subtraction of the diamagnetic contribution of the substrate. The loop exhibits a ferromagnetic character without reaching saturation. The presence of magnetic hysteresis indicates the ferromagnetic properties of the resulting films, while the substrate exhibits diamagnetic properties.

Since in the maximum magnetic fields achieved by a laboratory vibration magnetometer the magnetization did not reach saturation, the law of approach to saturation (LAS) was applied to estimate the value of saturation magnetization *M_S_*. The LAS determines the dependence of magnetization (*M*) on the applied field (*H*), where the applied field (*H*) is much greater than the coercive field (*H_C_*). LAS is usually used to describe magnetization in a region of strong magnetic field, where the rotation of the magnetic domain plays a significant role. According to [48] this value is determined by the expression:

(4) $$M=M_{s}\left[1-\frac{a}{H}-\frac{b}{H^{2}}\right]+kH$$

**Figure 10:**
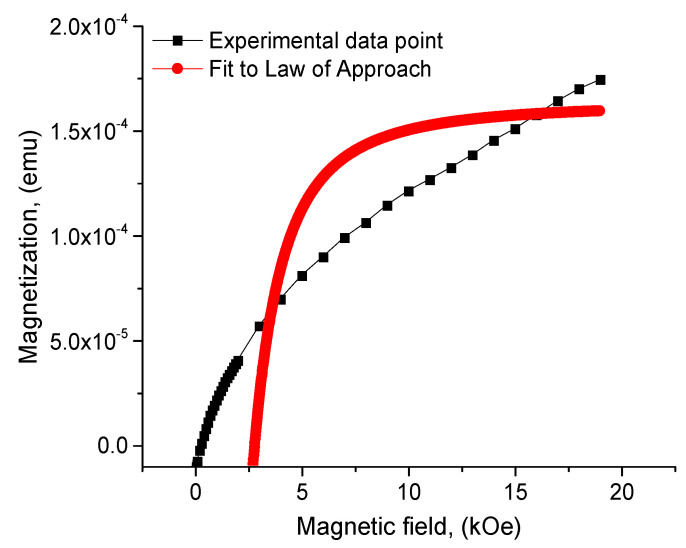
Fitting to the law of approach to saturation (LAS) for Bi_2_Fe_4_O_9_−BiFeO_3_.

A typical LAS fitting curve for the BiFeO_3_-Bi_2_Fe_4_O_9_ is shown in Figure 10. For this sample, the saturation magnetization, remanence, and coercive force were *M_s_* = 1.59 × 10^−4^ emu, *M_r_* = 1.33 × 10^−5^ emu and *H_C_* = 270 Oe, respectively and the *M_r_*/*M_s_* = 0.0833.

Note that a wasp-waist hysteresis loop was observed in the sample. Work by Lawrence H. Bennett and Edward Della Torre [49] examined how wasp-waist hysteresis loops arise in composite materials containing two ferromagnetic materials with different coercivities. Roberts and his colleagues, as well as Tauxe and his colleagues, used numerical simulations to determine the causes of this phenomenon. They found that the coexistence of materials with different properties, such as magnetic hardness and softness, anisotropy and coercive fields, or a mixture of single-domain and superparamagnetic particles can explain the appearance of the wasp-waist hysteresis loop [50,51,52].

## 4. Conclusions

By using the radio frequency cathode sputtering method of a ceramic target with a stoichiometric composition of BiFeO_3_ in an oxygen atmosphere, the nanocomposite films BiFeO_3_-Bi_2_Fe_4_O_9_ were obtained on a sapphire substrate Al_2_O_3_. Based on the results of the film analysis using X-ray structural analysis, Raman scattering, XPS, and atomic force microscopy, the structural parameters, topography, chemical composition of the films, concentration, and average sizes of the crystallites for each phase were determined. The results of the structural analysis demonstrated the formation of a two-phase structure, consisting of BiFeO_3_ (33.4%), Bi_2_Fe_4_O_9_ (63.4%), and a small amount of Bi_25_FeO_40_ (3.2%). The grain sizes, determined by AFM, were approximately 48 nm, and the root mean square roughness (Rms) of the film was 11 nm. The bandgap width was 1.9 eV for an indirect and 2.6 eV for a direct interband transition. The magnetic properties were characterized by a hysteresis loop resembling a wasp-waist, indicating the presence of magnetic anisotropy. The saturation magnetization, remanence, and coercive force were *Ms* = 1.59 × 10^–4^ emu, *Mr* = 1.33 × 10^–5^ emu, and *H_C_* = 270 Oe, respectively, with a *Mr/Ms* ratio of 0.0833. The results of the research can be useful in creating new materials for magnetoelectronics.

## Figures and Tables

**Table 1:** Structural parameters and average crystallite size.

Phase	Lattice Parameters			Crystallite Size (nm)	Phase Fraction (%)	R-Factors and χ^2^
	a	b	c			
BiFeO_3_	5.6010	5.6010	13.8125	14.9	33.4%	R profile—14.05 R expected—6.68 χ^2^—7.2
Bi_2_Fe_4_O_9_	7.8954	8.5891	6.0582	17.3	63.4%	
Bi_25_FeO_40_	9.8677	9.8677	9.8677	16.5	3.2%	

**Table 2:** Raman mode values (in cm^−1^).

BiFeO_3_	143	175	218	305	358	380	455	473	517	609
Bi_2_Fe_4_O_9_	232	280	328	423	546	630	752	807	-	-

## Data Availability

The data can be provided upon request to the corresponding author.
